# The healthcare needs and general practice utilization of people with acquired neurological disability and complex needs: A scoping review

**DOI:** 10.1111/hex.13640

**Published:** 2022-11-02

**Authors:** Stacey Oliver, Jacinta Douglas, Dianne Winkler, Christopher Pearce, Ella‐Rose Minter, Hannah K. Jarman, Megan Topping

**Affiliations:** ^1^ Department of Research and Innovation Summer Foundation Limited Victoria Blackburn Australia; ^2^ School of Allied Health, Human Services & Sport, Living with Disability Research Centre La Trobe University Melbourne Victoria Australia; ^3^ Aurora Primary Care Research Institute Victoria Melbourne Australia

**Keywords:** disability, general practice, health needs, primary care, utilization

## Abstract

**Background:**

For people with acquired neurological disabilities and complex needs, general practitioners (GPs) play an important role in health management and early intervention for the prevention of comorbidities and health complications. People with disability are a vulnerable group who need and have the right to, quality general practice services. It is therefore important to understand the health needs and service use of this group. The aim of this review was to identify the healthcare needs and general practice utilization of people with acquired neurological disabilities and complex needs.

**Methods:**

A scoping review methodological framework was utilized. Six databases (MEDLINE, PsycInfo, CINAHL, Scopus, Embase and the Cochrane Library) were searched. Articles were included if they reported on general practice service utilization of people with acquired neurological disabilities and complex needs aged between 18 and 65. Articles were required to be peer‐reviewed, written in English and published between 2010 and 2021.

**Results:**

Thirty‐one articles were included in the review. Studies originated from Canada (9), the United States (8), Australia (4), Switzerland (4), the United Kingdom (2), England (1), Norway (1), France (1) and Denmark (1). For many people, GPs were the main healthcare provider. People with disability consult multiple healthcare providers and navigate complex healthcare systems. Commonly presented healthcare needs were bladder, bowel and skin problems, pain and chronic pain, medication needs and mental health concerns.

**Conclusions:**

People with acquired neurological disabilities and complex needs were vulnerable to receiving suboptimal healthcare. The literature highlighted issues regarding the accessibility of services, the fragmentation of health services and inadequate preventative care. GPs were challenged to offer adequate disability‐related expertise and to meet the mental health needs of people with disability within time constraints.

**Patient and Public Involvement:**

This manuscript was prepared in collaboration with a GP, who is one of the authors. A person with lived experience of acquired neurological disability was engaged to check the alignment of the findings with their personal experience and provide feedback.

## 1 INTRODUCTION

People who have acquired neurological disabilities and complex needs have distinct healthcare needs and tend to experience increased health challenges and social vulnerability compared to the general population.^1^, ^2^ Acquiring a neurological disability and complex needs often occurs as a result of acquired brain injury (ABI) (including stroke), spinal cord injury (SCI), multiple sclerosis (MS) or cerebral palsy (CP). Such individuals share similar disability trajectories and experience profound functional impairment which results in interrelated needs.^3^ The breadth and severity of these needs coincide with increased health challenges and impacts life beyond the management of disease and symptoms^2^ resulting in a need for support in one or more life domains (e.g., mobility, self‐care, domestic life and/or self‐management)^4^ and potentially, comprehensive lifelong healthcare to support and maintain health and well‐being.^5^ Secondary morbidities such as chronic pain, bladder and bowel dysfunction, respiratory conditions and pressure ulcers often eventuate.^6^, ^7^ Additionally, health risk factors such as smoking, obesity and inactivity are substantially higher for this group compared to the general population.^1^ These issues often fall to primary care for initial management.^7^, ^8^, ^9^ Although primary care can involve several service providers (e.g., medical and allied health), encounters with general practitioners (GPs) are particularly important given their role as coordinators between patients and multiple healthcare providers.^8^, ^10^ However, the literature suggests that healthcare provided by GPs falls short of best‐practice care.^7^, ^11^

For people with acquired neurological disabilities and complex needs, early intervention and management of their conditions are particularly important as they can prevent the development of comorbidities and complications.^8^ High‐quality care provided by GPs has the potential to avoid unnecessary hospitalizations and improve health outcomes, individual function and well‐being over time.^10^ GPs focus on the holistic view of the patient, with a focus on prevention^12^ and understanding the whole patient.^13^ However, there are many constraints to service provision, including funding structure, time and persistence of the biomedical model.^14^, ^15^ Internationally, there has been an increase in GPs working as a part of multidisciplinary healthcare teams, on the assumption that interdisciplinary teams deliver higher quality services to patients.^16^, ^17^, ^18^ In the Australian context, GPs are increasingly part of a general practice care‐based team (with practice nurses) and are funded to support team‐based care.^19^ Similar team‐based models of care are implemented internationally, including in the United Kingdom, the Netherlands, the United States, Canada, Sweden, Spain and South Africa.^20^, ^21^

Despite the increased health needs of people with disability, evidence suggests that general practice does not serve people with complex disabilities as well as other patients.^22^ Challenges include insufficient building access or equipment, long waits for appointments and a lack of assistance with healthcare activities.^23^ Although higher levels of preventable health risk factors are evident for people with disability, studies have shown that people with disabilities are less likely to receive preventative care through GP services.^24^ Consequently, people with disability tend to use the emergency department more than their peers and often experience higher levels of unmet health needs.^11^, ^22^, ^23^ Given their increased health concerns and the importance of GP services for people with disability, it is important to understand the health needs and service use of this group.

The literature investigating health needs and healthcare service utilization of individuals with a disability is dominated by research specific to people with physical^25^ or intellectual disabilities.^26^, ^27^, ^28^ Substantially, fewer studies focus on individuals with acquired disabilities such as ABI, SCI, MS or CP. Of the limited research available, Dismuke et al.^29^ conducted a systematic review to examine health service utilization and cost in individuals with traumatic brain injury (TBI). Their findings revealed significant variation in healthcare costs and service utilization depending on the population and severity of TBI, with significant racial disparities in service utilization and access to primary care. McColl et al.^7^ identified common healthcare needs in their scoping review on primary care for people with SCI. Commonly raised issues in primary care included secondary complications such as bowel or bladder dysfunction and pain. The review also found significant unmet needs among people with SCI, including the need for information and specialized expertise. Most individuals with SCI reported being satisfied with the quality and accessibility of primary care; however, healthcare users had commonly developed complex strategies to navigate healthcare systems and services.^7^ The health needs and service utilization specific to general practice by people with acquired neurological disabilities and complex needs remain unclear.

People with acquired neurological disabilities and complex needs are a vulnerable group in need of reliable access to quality GP services over time. The increased and variable health concerns experienced by this cohort highlight the importance of maximizing the effectiveness of GP services for this group. Understanding the health needs of this cohort and their experiences with GP services can inform practice, policy, planning and funding and contribute to higher‐quality care. This scoping review sought to identify the healthcare needs and general practice utilization of people with acquired disabilities.

## 2 METHODS

The review utilized the scoping review methodological framework mapped by Arksey and O'Malley,^30^ combined with recent method enhancements.^31^, ^32^ This framework was deemed appropriate as the topic is large, and a scoping review offers a broader perspective regarding the topic of interest than a systematic review. The Prisma Extension for Scoping Reviews (PRISMA‐ScR): Checklist and Explanation informed the reporting of the method, study selection and results.^33^

### 2.1 Stage 1: Identifying the research question

The original objective of the review was to understand more about the current status of health issues faced by people with acquired neurological disabilities and complex needs, the primary care services available to this group, and whether services are utilized. However, it became apparent early during the screening that the literature spanning primary care was diverse and inconsistent due to international differences in terminology and healthcare systems. Due to these inconsistencies, and because the authors were primarily interested in understanding the health issues presented at general practice services for this group, a more specific question was developed: ‘What are the service and access needs regarding the general practice of people with acquired neurological disability and complex needs?’

For the purpose of this review, ‘people with acquired neurological disability and complex needs’ referred to people with acquired neurological disability and complex needs as the result of an ABI (including stroke), SCI, MS or CP.

### 2.2 Stage 2: Identifying relevant studies

The search strategy was developed by the research team, including content experts, in collaboration with an experienced research librarian (see Supporting Information: Appendix A: Search strategy table). Because the initial review aimed to identify the healthcare needs and primary care utilization more generally (e.g., allied health and community services), the search strategy included three broad concept headings: (1) population, (2) primary healthcare (including healthcare needs) and (3) utilization (e.g., of primary healthcare services). Systematic searching was conducted on six databases: MEDLINE, PsycInfo, CINAHL, Scopus, Embase and the Cochrane Library (trials only). Searches were limited to studies involving human participants published in English between January 2010 and October 2021. This timeframe was considered appropriate given the evolution of the healthcare system in the past decade. MeSH terms were added in Medline, CINAHL, PsycINFO and the Cochrane Library, and equivalent Emtree headings were added in Embase. Subsequent searching for relevant papers occurred via forwards and backwards citation searches of included articles.

### 2.3 Stage 3: Study selection

Following the inclusion criteria (see Supporting Information: Appendix B), a selection of 300 titles were double‐blind screened by three reviewers demonstrating 97% interrater agreement. Discussion to resolve conflicts occurred at each stage of screening. The remaining titles and abstracts were double‐blind screened by at least two of the primary reviewers. All study designs with extractable primary data were eligible for inclusion in the review (e.g., qualitative, quantitative, mixed‐methods, cross‐sectional, longitudinal). During the initial full‐text screening, consistency of terminology was identified as an issue across articles. Therefore, in line with the primary aim of the review, an additional exclusion criterion that outlined general practice services was developed. Specifically, if articles did not state that a provider of care services was a GP, Family Physician, Primary Care Physician, a Physician in a GPs office, Practice Nurse or Nurse Practitioner or if the setting or healthcare provider was not adequately described, they were excluded. Therefore, articles were included if the setting or healthcare provider was adequately described, and the reviewers could determine whether healthcare occurred at general practice. Additionally, an exclusion criterion for study design was required to ensure that reported study findings were specific to the population of interest (i.e., people with acquired neurological disability aged 18–65 years) and reported on general practice care in isolation from other types of care (e.g., secondary care). Articles were therefore excluded if results regarding general practice needs or utilization were unable to be isolated from other types of care (see Supporting Information: Appendix B: Exclusion criteria development, for a summary).

**Figure 1:**
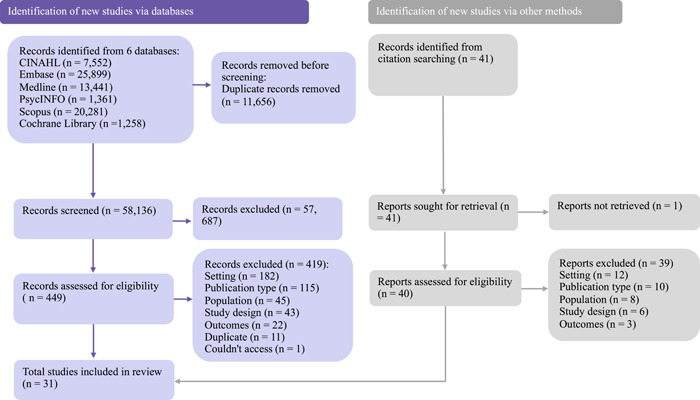
PRISMA flow diagram. Adapted from the PRISMA 2020 flow diagram template for systematic reviews.^32^

A total of 69,791 citations were initially retrieved. Covidence was utilized to remove 11,656 duplicates. The remaining 58,136 titles and abstracts were screened by three independent reviewers to determine the need for further evaluation of the full‐text article. After the full‐text screening of 449 articles, using the revised exclusion criteria, an additional 419 articles were excluded. One article was identified from forwards and backwards citation searches of the remaining 30 articles. Data were extracted from the final 31 articles. See Figure 1 for the full search process diagram.^34^

### 2.4 Stage 4: Charting the data

Data extraction was an iterative process conducted by the three primary reviewers and checked by co‐authors. Characteristics of included studies were extracted from 31 eligible articles to describe the population, setting, study design type and methods. Data were also extracted according to predetermined categories to summarize outcomes, results and findings in relation to the review question. Specifically, data relating to the utilization of (including access to) services, and the health needs presented at general practice services, were extracted. Where authors identified and reported gaps in the literature and research implications, the review authors charted this also. A narrative synthesis was conducted to collate the key information from the included articles.^30^ Keeping with the typical intent of a scoping review, the aim of this review was to provide a descriptive overview of the evidence in relation to the review question. In addition, due to the broad topic, a diverse range of research methodologies and data analysis techniques covered by the review (e.g., qualitative, quantitative, mixed‐methods, cross‐sectional, longitudinal), no quality appraisal of the included studies was performed.^31^, ^32^

### 2.5 Stage 5: Collating, summarizing and reporting results

**Figure 2:**
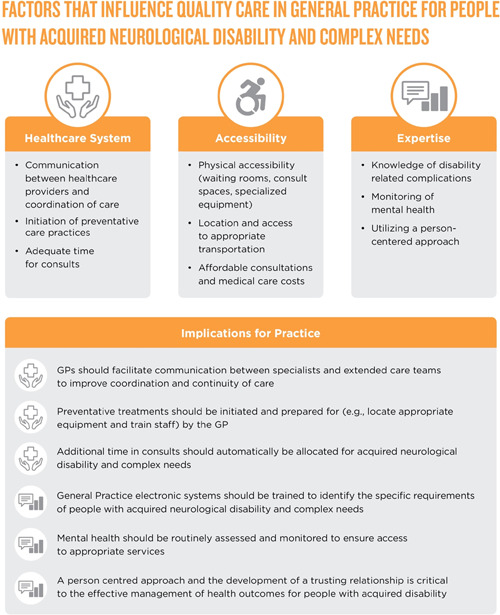
Summary of factors that influence quality care in general practice for people with acquired neurological disability and complex needs and implications for practice. Factors and implications are derived from the results of the scoping review.

The PRISMA‐ScR checklist guided the collating, summarizing and reporting of results.^33^ Key study and participant characteristics, as well as the main findings relating to the review question, are reported in Table 1. Section 3 presents collated findings from included articles for the healthcare utilization and healthcare needs of people with acquired neurological disabilities and complex needs. Quality of care received at general practice was identified as an issue when extracting the data. Findings regarding the quality of care are therefore also presented. Findings from the included articles and implications for practice were mapped onto Figure 2.

**Table 1:** Summary of study characteristics, participant characteristics and main findings

Study details	Methods	Main findings
Amsters et al.^35^; Australia; administrative data, longitudinal	*Population*: 193 participants; mean age = 43 years; male = 159 (82%); female = 34 (18%) *Disability*: SCI *Purpose*: To gather information specifically about the primary health care utilization patterns of people with SCI *Outcomes*: GP attendance	GP utilization was substantially greater for people with SCI compared with the general population and highest for young men with SCI, people with paraplegia and people with the highest impairment levels. GP use increased with age.
Barin et al.^36^; Switzerland; survey, longitudinal	*Population*: 1038 participants; median age = 48 years; male = 355 (26.2%); female = 683 (73.8%) *Disability*: MS (multiple sclerosis) *Purpose*: To examine disease‐specific and sociodemographic differences between MS patients in Neurological Care, General Practitioner Care or no Physician Care and to identify group differences and characteristics that may suggest care access barriers *Outcomes*: Health care use; trust in MC care provider	5% of participants reported only seeing a GP for healthcare; 89% of participants were in regular care by a neurologist; 6% reported seeing neither a GP or neurologist regularly. Compared to persons seeking only neurologist care, persons seeking only GP care were more likely to have Primary progressive MS and to have had an MS diagnosis for longer. 70% of people who saw a neurologist also saw a GP. Compared with their trust towards neurologists these patients seem to place less trust in their GPs.
Borg et al.^37^; Australia; survey; prospective cohort study	*Population*: 55 participants; mean age = 51; male = 42 (76.4%); female = 13 (23.6%) *Disability*: SCI *Purpose*: To examine the effects of health and rehabilitation service use, unmet need for services and service obstacles on health‐related quality of life and psychological well‐being after discharge from SCI rehabilitation *Outcomes*: Service usage; service obstacles; health‐related quality of life; mental health (depression, anxiety and stress)	Use of GP services was high at 6 and at 12 months postdischarge. There were no effects for use of GP on health‐related quality of life.
Brandon et al.^38^; Canada; semistructured interviews; cross‐sectional	*Population*: 16 participants with CP, 4 parents, 4 paediatricians, 4 primary healthcare physicians); mean age of participants with CP = 20; male = 4 (57.1%), female = 3 (42.9%) *Disability*: CP *Purpose*: To explore the experiences accessing or providing primary care services for young adults from the perspective of young adults with CP, parents, paediatricians and primary care physicians *Outcomes*: N/A	Inaccessible spaces impact on the quality of care provided to people with CP. Continual access to, and communication between multiple care providers, including family physicians, contributes to a successful transition from paediatric care to family physician care.
Chase et al.^39^; USA; survey; retrospective cohort study	*Population*: 75 participants with TBI; 2139 general population; mean age = 46.4; female = 2214 (100%) *Disability*: TBI *Purpose*: To investigate health factors related to primary health care, including screening and behavioural risk factors for women after TBI, and compare these with the general female population *Outcomes*: Preventative health measures; risk factors	Women with TBI were not disadvantaged compared with the general population with respect to breast and cervical cancer screening. Women with TBI had significantly poorer mental health compared to the general populations.
Chiu et al.^40^; USA; survey; cross‐sectional	*Population*: 3003 participants; mean age = 58.49; male = 514 (17.1%) female = 2489 (82.9%) *Disability*: MS *Purpose*: To evaluate priorities of patients with MS for their MS care *Outcomes*: MS course and treatment; MS care providers; locations of care; health care priorities	The healthcare provider primarily responsible for the participants' MS treatment and follow‐up was most often a neurologist (76.4%), followed by a general practice (7.2%), internist (2.3%) or nurse practitioner (2.1%). Participants wanted access to multiple providers and expressed frustration with the lack of knowledge about MS (i.e., its treatment, impact and interaction with other conditions) that nonspecialists had.
Crichton et al.^41^; England; registry data; cross‐sectional	*Population*: 648 participants aged 18–54; 671 participants aged 55–64; 2910 participants aged 65+; mean age not reported; male = 2125 (50.2%); female = 2104 (29.8%) *Disability*: Stroke *Purpose*: To compare access to care for younger adults to over 65s *Outcomes*: Indicators of care	Younger age was associated with increased GP contact at 3 months poststroke. Most younger stroke patients had equal, or greater, access to evidence‐based care when compared to older patients.
Dobos et al.^24^; USA; patient records; cross‐sectional	*Population*: 67 participants; mean age = 56.8; female = 67 (100%) *Disability*: MS *Purpose*: To describe preventive medical care for women with MS and to identify factors that may influence access to care *Outcomes*: Dates of annual primary‐care physician visits, mammography, Papanicolaou smear, colonoscopy and bone density scans	Women with severe MS are vulnerable to significantly decreased access to preventive care.
Gemperli et al.^42^; Switzerland; survey; cross‐sectional	*Population*: 492 participants; mean age = 55; male = 350 (71.1%); female = 142 (29%) *Disability*: SCI *Purpose*: To identify the care‐seeking behaviour of persons with SCI for various healthcare providers and circumstances when required care was not received *Outcomes*: Utilization of health care providers; failure to receive health care support	Individuals with SCI are frequent users of medical services. GPs and physiotherapists were the main healthcare providers for persons with SCI. Instances where care was required but not received were more likely for people with migration background.
Gemperli et al.^43^; Switzerland; survey; cross‐sectional and longitudinal subgroup	*Population*: 1294 cross‐sectional participants; mean age = 56.4; male = 917(70.9%); female = 377 (29.1%); 221 longitudinal participants; mean age = 59.9; male = 161 (72.9%); female = 60 (27.1%) *Disability*: SCI *Purpose*: To understand the use of outpatient healthcare providers by individuals with chronic spinal cord injury *Outcomes*: Utilization of healthcare providers	The healthcare provider was most often visited was a general practitioner (82%), followed by the physiotherapist (63%), and then the dentist (62%). Participants used multiple healthcare providers.
Hagen et al.^44^; Norway; survey; cross‐sectional	*Population*: 105 participants; mean age = 49.5; male = 84 (80%); female = 21 (20%) *Disability*: SCI *Purpose*: To examine how satisfied patients with traumatic SCI are with their general practitioners *Outcomes*: Quality of life; patient satisfaction	Patients with SCI were more satisfied with their GP than the Norwegian population. Patients with incomplete SCI were least satisfied with their GP. Patients with anxiety and/or depression were more likely to report low satisfaction with their GP.
Hamilton et al.^45^; USA; survey; cross‐sectional	*Population*: 142 participants; mean age = 40.8; male = 92 (65%); female = 50 (35%) *Disability*: SCI *Purpose*: To describe the utilization, accessibility and satisfaction of primary and preventative healthcare services of community‐dwelling individuals with SCI *Outcomes*: Healthcare utilization; healthcare access; preventative health information; satisfaction with current healthcare delivery	The majority of participants (79%) see a primary care physician, with the primary reason for visit being genital/urological (15%). People with SCI experience accessibility barriers at their primary care physician's office. The majority of participants (66%) reported that their primary care physician was knowledgeable about their SCI needs.
Jones et al.^46^; USA; survey; cross‐sectional	*Population*: 715 participants; mean age = 42.1; male = 216 (30.6%); female = 499 (69.8%) *Disability*: MS *Purpose*: To quantify the relationship between increased disability and healthcare utilization, healthcare cost, quality of life, ability to work and productivity while at work among patients with MS *Outcomes*: Healthcare utilization	Compared with patients with no relapse, those with relapse made significantly more visits to a primary care physician.
Lakhani et al.^47^; Australia; survey; cross‐sectional	*Population*: 266 participants; mean age = 62.43; male = 140 (52.6%); female = 116 (43.6%) *Disability*: Spinal cord damage *Purpose*: To identify factors contributing to GP identification and retention *Outcomes*: GP identification; GP retention	GPs were mostly identified by having seen the GP before injury or by trying multiple GPs until finding one best suits their needs. Participants with spinal cord damage retained their GP based on GP knowledge about their condition, the physical accessibility of the service and trust.
Lala et al.^48^; Canada; survey; cross‐sectional	*Population*: 1137 participants with pressure ulcer; mean age = 47.8; male = 279 (73%); female = 102 (27%).756 participants without pressure ulcer; mean age = 48.6; males = 527 (70%); females = 229 (30%) *Disability*: SCI *Purpose*: To describe the impact of pressure ulcers on the ability to participate in daily and community activities, health care utilization and overall quality of life in individuals living with SCI *Outcomes*: Healthcare utilization	Individuals with pressure ulcers visited GPs and nurses more than physiatrists or wound care nurses/specialists to assist in managing their pressure ulcer. Pressure ulcers were found to increase visits to the GP.
Laursen et al.^49^; Denmark; registry data; cohort study	*Population*: 434 participants with TBI; mean age = 40; male = 303 (70%); female = 130 (30%); 100 participants with SCI; mean age = 34; male = 69 (69%); female = 31 (31%) *Disability*: TBI, SCI *Purpose*: To estimate the health service use over 9 years after acquiring, SCI, pelvic fracture and compare with noninjured *Outcomes*: number of services from general practitioners	People with TBI and SCI had increased GP following injury compared to a matched reference group during the follow‐up period. There were no differences in the mean use of GP between people with TBI and SCI.
Lank et al.^50^; USA; population‐based data; cross‐sectional	*Population*: 151 participants without routine primary care provider; mean age = 56; male = 95 (62.9%); female = 56 (37.1%); 512 participants with a routine primary care provider; mean age = 56; male = 283 (55.2%); female = 229 (44.9%) *Disability*: Stroke *Purpose*: To determine whether having a primary care physician at the time of first stroke is associated with a lower risk of stroke recurrence and mortality than those who do not have a primary care physician *Outcomes*: Primary care physician use; stroke recurrence	Having a primary care physician at the time of first stroke was associated with lower rates of stroke recurrence.
Lofters et al.^51^; Canada; survey and retrospective chart review; cross‐sectional	*Population*: 60 participants with SCI; mean age = 54; male = 38 (63%; female = 22 (37%); 15 physicians; age not reported; male = 8 (53.3%); female = 7 (46.6%) *Disability*: SCI *Purpose*: To determine patterns of preventive care for patients with SCI in a primary care setting and determine physicians' level of comfort with providing primary care to patients with SCI *Outcomes*: Influenza vaccinations; general physicals; bone mineral density tests; physicians' level of comfort	People with SCI had low cancer screening rates, and many had no record of a flu shot. Physicians tended to have few patients with SCI, and co‐managed care with specialists. GPs were comfortable managing mental health issues, urinary tract infections, skin concerns, providing immunizations and monitoring blood pressure, but most were not comfortable managing autonomic dysreflexia.
McMillan et al.^52^; Canada; semistructured interviews; cross‐sectional	*Population*: 20 family physicians; mean years in practice = 16; male = 12 (60%); female = 8 (40%) *Disability*: Severe mobility impairments *Purpose*: To explore family physicians' perspectives on primary care for individuals with mobility impairments to identify and better understand the challenges that prevent equitable service delivery to this group of patients *Outcomes*: N/A	GPs are challenged to meet the health needs of individuals with mobility impairments, particularly wheelchair users. A number of gaps in care were identified, including a lack of specialized equipment, scarcity of preventive care, lack of availability of and access to standardized care tools and a lack of staff training or familiarity with transfer equipment.
McMillan et al.^53^; Canada; mixed‐methods; cross‐sectional	*Population*: 12 family physicians; 4 participants practicing for 10–19 years; 2 participants practicing 20–29 years; 1 participant practicing for >30 years; male = 7 (58.3%); female = 5 (41.7%) *Disability*: SCI *Purpose*: To gather primary care health provider and rehabilitation specialists' perspectives on why challenges in health care for people with SCI remain *Outcomes*: Barriers to optimal care, potential solutions	Lack of education for GPs, fragmentation of community resources and co‐ordination upon hospital discharge were highlighted as barriers to optimal care.
Mesidor et al.^54^; Canada; administrative data; longitudinal	*Population*: 1426 participants; mean age at diagnosis = 32.4; male = 340 (23.8%); female = 1086 (76.2%) *Disability*: MS *Purpose*: To study the association between age at diagnosis and the use of health services for MS *Outcomes*: Number of MS‐related visits to a GP, neurologist, emergency room, number of days of hospitalization	The rate of GP use was higher in the year of MS diagnosis compared to the other periods. People who were diagnosed after 29 years of age had a higher rate of visits to a GP during the 9th year following MS diagnosis.
Methley et al.^55^; UK; semistructured interviews; cross‐sectional	*Population*: 24 participants with MS; mean age not reported; male = 5 (20.8%); female = 19 (79.2%); 12 general practitioners; male = 5 (41.6%); female = 7 (29.1%); 3 participants aged; mean age not reported *Disability*: MS *Purpose*: To investigate the healthcare experiences and experiences with primary and secondary care professionals of people with MS *Outcomes*: N/A	Positive experiences of healthcare centred around timely access to services, relational continuity of care and person‐centred interactions.
Methley et al.^56^; UK; semistructured interviews; cross‐sectional	*Population*: 24 participants with MS; mean age not reported; male = 5 (20.8%); female = 19 (79.2%); 12 general practitioners; mean age not reported; male = 5 (41.6%); female = 7 (29.1%) *Disability*: MS *Purpose*: To explore perspectives and experiences of people with MS and healthcare professionals of mental health support for MS in the United Kingdom *Outcomes*: N/A	GPs are a central figure in the management of mental health needs in MS.
Petrin et al.^57^; Canada; focus group and 1:1 interviews; cross‐sectional	*Population*: 38 focus group participants; 10 interview participants; mean age of total participants = 49.6; male = 16 (33%); female = 32 (67%) *Disability*: MS *Purpose*: To investigate the healthcare access experiences of Ontarians with MS *Outcomes*: N/A	A renewed effort to promote patient‐centred care and a biopsychosocial approach may improve the healthcare access experiences of persons with MS and reduce service avoidance.
Petrin et al.^58^; Canada; focus group and 1:1 interviews; cross‐sectional	*Population*: 38 focus group participants; 10 interview participants; mean age of total participants = 49.6; male = 16 (33%); female = 32 (67%) *Disability*: MS *Purpose*: To align the experiences of persons with MS in accessing healthcare services with the stages of the Candidacy Framework *Outcomes*: N/A	Neurologists were most often reported as being the main source of MS care (*n* = 29, 60.4%) followed by Family physicians (*n* = 15, 31.3%). Persons with MS described a complex decisional process related to the Identification of Candidacy which was pivotal to their access experience.
Ronca et al.^59^; Switzerland; survey; cross‐sectional	*Population*: 492 participants; mean age = 57; male = 349 (71%); female = 143 (29%) *Disability*: SCI *Purpose*: To identify barriers to access healthcare services and reveal determinants of satisfaction with healthcare services in people with SCI *Outcomes*: Availability and quality of healthcare services for secondary health conditions; satisfaction with fulfilment of healthcare needs, preferences for care	Reported satisfaction with quality of primary care services was low when access to short and long‐distance transportation was insufficient. A lack of accessibility to long distance transportation was strongly associated with a perception of unmet SCI‐related care needs. People with incomplete paralysis and pain consistently rated the fulfilment of care needs associated with SCI less favourably.
Roux et al.^60^; France; administrative data; cross‐sectional	*Population*: 112,415 participants; median age = 46; male = 32,680 (29.1%); female = 79,735 (70.9%) *Disability*: MS *Purpose*: To describe use of care in the French population of people with Multiple Sclerosis *Outcomes*: Use of healthcare services	GPs were the most frequently visited healthcare provider. People with MS visited GPs significantly more than the general population. The presence of co‐morbidities was associated with higher GP use.
Stillman et al.^64^; USA; survey; cross‐sectional	*Population*: 432 participants; mean age not reported; male = 156 (35.9%); female = 276 (64.1%) *Disability*: Physical disability (neurological and rheumatological conditions) *Purpose*: To describe healthcare utilization among wheelchair users and characterize barriers encountered when attempting to obtain access to health care *Outcomes*: Healthcare utilization; receipt of preventive services; physical barriers encountered at outpatient facilities; satisfaction with care	Wheelchair users face persistent barriers to care, may receive less than thorough physical evaluations, receive fewer screenings for cervical cancer and largely believe they receive incomplete care.
Stillman et al.^61^; USA; survey; cross‐sectional	*Population*: 108 participants; mean age = 48; male = 60 (55.6%); female = 48 (44.4%) *Disability*: SCI *Purpose*: To identify health care utilization and preventative care screening utilization, as well as barriers to accessing primary and speciality care services for people with SCI *Outcomes*: Healthcare utilization; barriers encountered when accessing healthcare facilities; receipt of routine care and preventative screenings	Barriers to care included inaccessible examination and lacking accessible examination tables, proper transfer aids and staff capable of assisting with patient transfers. People with SCI receive less than thorough care and many expressed frustrations that their primary care providers were not knowledgeable of the impact of SCI on their overall health. Few people with SCI had received screenings for colorectal, cervical and breast cancers. More than half of the participants (53.7%) were either satisfied or very satisfied with the examination they had received from their primary care provider.
Toor et al.^62^; Canada; survey; retrospective cohort study	*Population*: 105 participants; mean age = 35.2; female = 105 (100%) *Disability*: TBI *Purpose*: To assess long‐term healthcare service utilization and satisfaction with healthcare services among women with TBI, examine barriers that prevent with TBI from receiving care when needed; and understand the perceived supports available for with TBI *Outcomes*: Services utilization; satisfaction with and quality of services; barriers to receiving care; perceived social support	Compared with women without TBI, women with TBI reported using more family physician and community health services. Women with TBI reported that they did not receive care when needed (40%), particularly for emotional/mental health problems. Significantly more women with TBI reported financial and structural barriers. There were no significant differences in reported satisfaction with services between women with and without TBI.
Zhou et al.^63^; Australia; survey; nationally collected data; cross sectional.	*Population*: 11,662 participants; mean age not reported; male = 5715 (49%); female = 5947 (51%) *Disability*: Head injury, stroke or brain damage; core activity limitation; sight, hearing and speech disability; physical disability; intellectual disability; psychological disability *Purpose*: To quantify the role of disability status, types of disability and severity of disability in their respective relationship to psychological distress in adults, and further explore the mediating effect of health service use on the disability‐distress association *Outcomes*: psychological distress, annual visits to general practitioner	For the mediating effect of different health service use, GP services demonstrated the greatest decrease of the effect of disability on high psychological distress. GPs hold an important place to deal with the disabling and/or secondary clinical conditions as potential stressors.

### 2.6 Stage 6: Lived experience consultation

After the results had been collated and summarized, a person with lived experience of an acquired neurological disability was engaged to check the alignment of the findings with their personal experience.^31^ Overall, the consultant's perspective indicated most of the findings were congruent with their experience.

## 3 RESULTS

### 3.1 Participant characteristics

The 31 papers included in the review shared several common characteristics. Participants with moderate‐severe disabilities were included in all selected studies. As can be seen in Table 1, 11 papers reported on participants with SCI, 10 on participants with MS, 2 on participants with TBI, 2 on participants with stroke, 1 on participants with CP, 1 on participants with spinal cord damage and 4 on participants with multiple disabilities. Participants' comorbidities were reported in eight studies. The number of participants in the studies ranged from 16 to 3003. Most papers reported on a population with a mean age over 40 years. One study reported on participants with a mean age of 20 years. Of the studies included, 20 reported solely the perspective of people with disability.^35^, ^36^, ^37^, ^39^, ^40^, ^42^, ^43^, ^44^, ^45^, ^47^, ^48^, ^49^, ^50^, ^57^, ^58^, ^59^, ^61^, ^62^, ^63^, ^64^ Four reported on the perspective of the person with a disability, as well as the GPs' perspective^38^, ^46^, ^55^, ^56^ and one paper reported on the perspective of the person with disabilities, alongside administrative data.^54^ Two papers reported only on the perspective of the GP^52^, ^53^ and one reported on the GPs' perspective alongside administrative data.^51^ The remaining three papers reported only on administrative data.^24^, ^41^, ^60^

### 3.2 Study characteristics

Table 1 provides an overview of the characteristics, outcomes assessed and main findings of the studies. Of the studies included, 6 used a qualitative methodology, 24 were quantitative and 1 used mixed methods. Nine studies originated from Canada, eight from the United States, four from Australia, four from Switzerland, two from the United Kingdom, one from England, one from Norway, one from France and one from Denmark.

#### 3.2.1 Synthesis

The following data synthesis should be considered in the broad context of healthcare systems and sociocultural backgrounds that have influenced the findings of this literature.

### 3.3 Utilization of general practice services

Findings regarding the frequency of GP use consistently indicated that people with disabilities are more frequent users of GPs compared to the general population,^35^, ^60^, ^62^ particularly in the first 1–3 years following discharge from hospital or diagnosis.^37^, ^49^, ^54^ Utilization of GP services increased with disability severity or complexity.^36^, ^46^, ^60^ Mixed findings regarding age and GP use were evident. Younger age of stroke was associated with high GP use,^41^ whereas GP use increased with age in people with SCI and people with MS.^35^, ^54^ One study found gender differences regarding service use. Specifically, young men with SCI sought help from GPs at a greater rate than females with SCI and the general population.^35^ Interestingly, in a study of people with SCI, Borg et al.^37^ found no relationship between the frequency of GP use and self‐reported quality of life.

Utilization of GP services differed by disability type. Although people with SCI and TBI used GP services more frequently for 1–3 years postinjury, longer‐term service use was significantly different between groups. For people with SCI, the use of GP services remained at an increased level for 9 years postinjury, but for those with TBI, GP service use returned to baseline level at 4 years postinjury.^49^ Jones et al.^46^ found that people who experienced MS relapse made significantly more visits to their GP in the 12 months before relapse compared to those not experiencing a relapse. Lank et al.^50^ found that 5‐year stroke recurrence risk was 75% greater for those without a GP at the time of the first stroke compared to those who had a GP at the time of the first stroke, indicating that having a GP is associated with a lower risk of stroke recurrence.

### 3.4 Utilization of healthcare providers

Although it was consistently found that people with disability are high users of GP services, mixed findings were apparent regarding participants' main healthcare providers. Multiple studies found that GPs were the main source of healthcare for people with disabilities.^42^, ^43^, ^44^, ^48^, ^55^, ^60^ In particular, people with SCI reported visiting GPs more than rehabilitation specialists, wound care nurses or specialists to assist in managing pressure ulcers.^48^ GPs were central figures in the management of mental health needs for people with MS due to their continuing close relationships with patients and their ability to manage pharmacological treatments.^55^ However, neurologists were also commonly found to be primary healthcare providers for people with MS in the United States, Canada and Switzerland.^36^, ^40^, ^57^, ^58^

People with disabilities consistently utilized multiple healthcare providers^36^, ^38^, ^40^, ^42^, ^43^, ^45^, ^46^, ^48^, ^49^, ^57^, ^58^, ^60^, ^61^, ^64^ and fragmentation of services was an issue.^38^, ^40^, ^52^, ^53^, ^55^, ^56^, ^58^ Poor continuity of care was commonly mentioned as a barrier to receiving quality healthcare by both people with disability^38^, ^40^, ^55^, ^58^ and by GPs.^52^, ^53^, ^55^ People with a disability wanted increased continuity of care among healthcare providers,^38^, ^40^, ^55^, ^58^ and GPs highlighted that patients often moved between healthcare providers without anyone being primarily responsible for their overall care.^52^, ^53^

Compared to those with less severe disabilities, people with complex disabilities more often utilized multiple healthcare providers.^38^, ^40^, ^51^ Providers frequently visited in combination with GPs were physiotherapists, urologists, physiatrists, neurologists, dentists or dental hygienists and for females, obstetricians or gynaecologists.^42^, ^43^, ^45^, ^48^, ^49^, ^55^, ^57^, ^58^, ^60^, ^62^, ^63^

### 3.5 Access to GP services

Challenges with accessibility in GP offices have an impact on the quality of care received.^38^, ^39^, ^45^, ^52^, ^57^, ^58^, ^59^, ^61^, ^62^, ^64^ Research originating from the United States and Canada report issues with physical accessibility for people with SCI and severe mobility impairments.^45^, ^52^, ^61^, ^64^ Examination rooms frequently lacked accessible doorways, examination tables, accessible scales, proper transfer aids and staff capable of assisting with patient transfers.^45^, ^52^, ^61^, ^64^ Additionally, multiple studies found that accessible equipment could not be located or operated by GPs or available staff members.^52^, ^61^, ^64^ In a qualitative study, GPs reported that the inability to conduct a thorough physical examination had resulted in patients either not being examined or being referred to the local emergency department or specialists for issues that theoretically should have been managed in primary care.^52^

Challenges with access to healthcare due to location and transportation issues were also identified.^37^, ^42^, ^52^, ^53^, ^57^, ^58^, ^59^, ^62^ Ronca et al.^59^ found that insufficient access to appropriate transportation was a barrier to accessing GP services for people with SCI. Specifically, the risk of experiencing unmet SCI‐related healthcare needs was almost three times higher for those with problems accessing transportation than for those without. Another study found that compared to the general population, women with TBI experienced significantly more structural barriers when accessing GP offices^62^ and transportation barriers were the main reason for not receiving needed care.^37^, ^62^ Indeed, Methley et al.^55^ highlighted that when people with MS were located close to their GPs and perceived the location as relatively easy to access, the service was highly utilized. Issues related to transportation to GP offices, including no access to, or difficulty accessing suitable transportation, were a concern of GPs in both urban and rural contexts.^52^

Financial issues also affected access to GPs.^37^, ^39^, ^42^, ^57^, ^58^, ^62^ Medical care costs were the primary barrier to access.^39^ Other costs such as transportation, parking fees and accommodation (if required) were also commonly identified as barriers to accessing general practice services.^37^, ^57^, ^58^

### 3.6 Health needs presented at general practice

A limited number of studies reported specifically on the health needs presented by people with acquired neurological disabilities and complex needs at GP offices.^42^, ^44^, ^45^, ^48^, ^56^, ^59^ Generally, GP services were sought for acute care needs.^42^, ^52^, ^58^ Health needs included bladder, bowel and skin problems, pain or chronic pain, mental health concerns and medication requirements.^42^, ^44^, ^45^, ^48^, ^56^, ^59^ Health needs for which care was required, but not received or sought, were bladder problems, bowel problems, chronic pain and medication requirements.^42^, ^59^ In a qualitative study, Lofters et al.^51^ investigated GPs' comfort level in managing the specific health needs of people with SCI. All GPs reported some level of comfort in treating mental health issues, urinary tract infections and skin concerns, providing immunizations and monitoring blood pressure. However, comfort levels for managing spasticity, respiratory issues and autonomic dysreflexia were low.

GPs were challenged to meet the mental health needs of people with disabilities^44^, ^56^, ^62^ and satisfaction with GPs' ability to treat mental health issues was also low.^44^, ^56^ Toor et al.^62^ found that 40% of women with TBI reported not receiving postinjury care when needed, particularly for emotional or mental health problems, which was significantly more than women without TBI. In a qualitative study, GPs described that due to a complex interplay of mental health and physical well‐being in people with MS, doctors often do not view mental health symptoms as distinct conditions that require support.^56^ Despite seeing high levels of depression and anxiety and playing a central role in the management of mental health, GPs described resisting pathologizing symptoms of low mood and utilizing social explanations of depression onset and maintenance (e.g., relationship breakdowns or the stigma attached to physical symptoms such as incontinence).^56^ Interestingly, in a comparison of health services, Zhou et al.^63^ found that the use of GP services demonstrated the greatest reduction in disability‐related distress for people with a head injury, stroke or brain damage (44%), people with a physical disability (28%) and for people with sight, hearing and speech disability (12%).^63^ These findings indicate that GPs in the primary healthcare setting occupy an important place in dealing with disability‐related stressors and psychological distress.

### 3.7 Quality of care provided at general practice

Through documenting healthcare needs and general practice utilization, factors that influence quality care in general practice were identified. The quality of care provided by GPs was a common issue raised.^44^, ^48^, ^52^, ^59^, ^61^, ^64^ People with more complex and severe disabilities were consistently less likely to receive comprehensive care^44^, ^48^, ^52^, ^59^, ^61^ and were less satisfied with the quality of care received from GPs.^44^ However, several studies found people with acquired neurological disability and complex needs were satisfied with the quality of care received from GPs,^45^, ^61^, ^62^, ^64^ often despite reporting receiving incomplete care.^61^, ^64^ Petrin et al.^57^ found that participants' perceptions of a healthcare encounter were strongly influenced by whether they received patient‐centred care. When patient‐centred care was not received, multiple negative outcomes followed, including feeling disregarded, invalidated and dismissed.

The frequency and quality of preventative care provided by GPs were often reported as suboptimal. Numerous studies found that people with acquired neurological disabilities and complex needs received decreased access to preventive care, including low rates of cancer screening, influenza vaccinations and bone mineral density testing.^24^, ^51^, ^52^, ^58^ Preventive care was more frequently administered for simple procedures such as influenza and pneumococcal vaccinations, than for more complex preventive care screenings that require preparation or special equipment (such as for colorectal, cervical and breast cancer screening) when administered for wheelchair users or people with SCI.^61^, ^64^ Reasons for not receiving preventive care were lack of physician recommendation or GP practices located in inaccessible buildings.^64^ However, one study found that women with TBI were not disadvantaged compared with the general population with respect to breast and cervical cancer screening.^39^

Knowledge of disability‐related complications was also highlighted as an issue.^40^, ^44^, ^47^, ^52^, ^53^, ^57^, ^61^, ^64^ Many participants expressed frustration and concerns that their GPs did not understand the impact their disability had on their overall health or its related health complications.^40^, ^44^, ^52^, ^61^, ^64^ Disability‐specific knowledge, along with physically accessible services and trust, were important considerations for those with spinal cord damage when choosing and maintaining a GP.^47^ Additionally, GPs expressed difficulties in providing care for people with acquired neurological disabilities and complex needs.^38^, ^52^, ^53^, ^55^ These difficulties included restrictions on the time available to manage complex needs and have the required knowledge for treatment options.^38^, ^52^, ^53^, ^56^, ^58^ One study found that people with acquired neurological disabilities tended to trust specialists more than GPs, in particular with knowledge related to their disability.^36^

## 4 DISCUSSION

The aim of this scoping review was to document healthcare needs and general practice utilization of people with acquired neurological disabilities and complex needs, as reported in peer‐reviewed literature. People with acquired neurological disabilities visit GPs frequently,^35^, ^37^, ^39^, ^45^, ^60^, ^61^, ^62^ particularly in the years following discharge from the hospital,^37^, ^49^, ^54^ and in the case of those with more complex or severe disabilities.^36^, ^46^, ^60^ People with acquired neurological disability commonly present to GPs with acute care needs, including bladder, bowel and skin problems, pain and chronic pain, medication needs and mental health concerns.^42^, ^52^, ^58^ Despite the increased use of GP services, people with acquired neurological disabilities were consistently less likely to receive the full range of care compared to the general population^44^, ^48^, ^52^, ^59^, ^61^, ^64^ and people with more complex and severe disabilities were consistently less likely to receive complete care.^44^, ^48^, ^52^, ^59^, ^61^ As summarized in Figure 2, findings highlight that systemic,^38^, ^40^, ^52^, ^53^, ^55^, ^58^ access^37^, ^38^, ^39^, ^42^, ^45^, ^52^, ^53^, ^57^, ^58^, ^59^, ^61^, ^62^, ^64^ and expertise^40^, ^44^, ^47^, ^52^, ^53^, ^57^, ^61^, ^64^ factors all influence quality care in general practice. Figure 2 also presents the recommendations for practice derived from the results of this scoping review.

Healthcare system factors that were found to contribute positively to quality care in general practice included continuity of care, initiation of preventive healthcare practices and having adequate time for consults. For people with acquired neurological disabilities, coordination of healthcare is often complex and left to the individual.^38^, ^40^, ^52^, ^53^, ^55^, ^58^ In addition, people with acquired neurological disabilities and complex needs commonly utilize multiple healthcare providers alongside general practice services.^36^, ^38^, ^40^, ^42^, ^43^, ^45^, ^46^, ^48^, ^49^, ^57^, ^58^, ^60^, ^61^, ^64^ This fragmentation of services and lack of continuity challenges the provision of quality care for people with acquired neurological disabilities and, in some cases, contributed to their negative health outcomes.^38^, ^40^, ^52^, ^53^, ^55^, ^57^ Policy and practice responses around the world are being developed to assist patient access to quality multidisciplinary team‐based primary care.^65^ Increasingly evidence suggests that collaboration within healthcare services does not work as intended^53^, ^66^, ^67^ and general practice services should facilitate communication between specialists and extended care teams to maximize the quality of care. This role is increasingly played by practice nurses who work closely with GPs.^17^ Findings from this review further highlight the need for integration and collaboration between healthcare providers.

This review also demonstrates that individuals with an acquired neurological disability and complex needs often receive suboptimal preventive care^24^, ^51^, ^52^, ^58^ and experience multiple barriers to preventative treatments^52^, ^61^, ^64^ echoing previous research finding that adults with intellectual disability are less likely to receive preventative care compared to the general population.^27^ Screening methods are crucial to prevention, early diagnosis and treatment.^11^, ^22^, ^23^ To maintain preventive care practices, it is recommended that GPs proactively prepare for the delivery of relevant preventive treatments (e.g., locate appropriate equipment and train staff). In addition, general practice electronic systems should be modified to identify the specific requirements of patients with different types of disabilities. Electronic medical record systems could introduce prompts to support GPs to ask more relevant questions and monitor for common secondary complications. Additionally, time constraints are a barrier to GPs provision of quality care.^38^, ^52^, ^53^, ^55^ People with acquired neurological disabilities and complex needs require additional consultation time for those needs to be fully explored.

Accessibility factors influencing quality care in general practice included physical accessibility, access to specialized equipment, transport and affordable medical costs. In line with previous literature,^5^, ^68^ this review found that physical accessibility remains a barrier to receiving comprehensive health and preventive care from GPs.^38^, ^39^, ^45^, ^52^, ^57^, ^58^, ^59^, ^61^, ^62^, ^64^ Furthermore, it suggests that improving accessibility may prevent unnecessary use of emergency services and admissions to hospitals.^52^, ^61^, ^64^ Accessibility of general practice facilities does vary internationally, likely due to the implementation and adoption of national accessibility policies or standards. The research highlighting physical accessibility barriers in the current review originates from the United States and Canada. The Americans with Disabilities Act requires that healthcare entities provide full and equal access to people with disabilities, yet there is growing consensus that the standards are not appropriately enforced and are also inadequate to achieve equal access.^69^ Furthermore, a recent environmental scan of the accessibility legalization, standards and guidelines across Canada found that accessibility varied throughout the country due to diverse standards and guidelines in different jurisdictions.^70^ Hence, physical accessibility may remain an issue in some countries due to standards not being adopted (e.g., voluntary standards rather than legally required standards) or implemented correctly, lack of monitoring and enforcement (i.e., due to no governing body), or standards not adequately meeting the needs of people with acquired neurological disability and complex needs.

The cost of general practice services and financial restrictions also negatively impact access to GPs.^37^, ^39^, ^42^, ^57^, ^58^, ^62^ Complex system factors, such as a country's health and insurance schemes, as well as societal inequities contributing to financial difficulties (e.g., lower wages, reduced employment opportunities), contribute to the affordability of general practice services for people with acquired neurological disability.^37^, ^62^ Given early identification of health conditions can improve health outcomes^41^ and reduce long‐term healthcare costs,^71^ the financial obstacles experienced by people with disability when accessing general practice services requires further exploration.

Transportation (e.g., travel time, distance and cost) was frequently a barrier to accessing GP services^37^, ^42^, ^52^, ^53^, ^57^, ^58^, ^59^, ^62^ and associated with poorer health outcomes for people with acquired neurological disability and complex needs.^37^, ^62^ Access to services is a known determinant of health.^5^, ^68^ Lack of access to healthcare appointments results in missed opportunities for support and treatment placing people at risk of further complications.^68^ Findings from this scoping review are consistent with previous literature finding physical access to healthcare premises is problematic for people with a range of disabilities.^25^ Future research should investigate the effectiveness of short‐term solutions to transportation and location barriers, such as GPs accommodating afternoon appointments, home visits and telecare. Longer‐term solutions to ensure people with acquired neurological disabilities have adequate access to GP services include better collaboration between policymakers, urban planners, architects, advocates and healthcare providers.

The expertise of healthcare providers and knowledge of disability‐related challenges has previously been highlighted as a barrier for people with disability receiving quality care.^7^, ^72^ Factors related to expertise include GPs' knowledge of disability‐related complications, ongoing monitoring of mental health and using a person‐centred approach.^40^, ^44^, ^47^, ^52^, ^53^, ^57^, ^61^, ^64^ As highlighted in this review, people with acquired neurological disabilities and complex needs can be high users of GP services and present multiple needs.^35^, ^37^, ^39^, ^45^, ^60^, ^61^, ^62^ This requires that GPs understand an extremely wide range of conditions to address the complexity of peoples' needs in a restricted amount of time.^38^, ^52^, ^53^, ^55^, ^58^ Expertise of healthcare providers appears to be a consistent barrier to receiving quality care for people with disability.^25^, ^27^ Further research is needed to identify the unique health needs presented at general practice across different disability groups.

Despite the high prevalence of mental health issues in people with acquired neurological disabilities,^73^, ^74^, ^75^ mental health concerns were under‐treated.^44^, ^56^, ^62^ GPs should ensure specific resources and mechanisms (e.g., access to special services, support from regional health services) are available to people with acquired neurological disabilities and complex needs. It is increasingly recognized that one primary care provider cannot meet all the needs of people with complex health problems.^2^, ^65^ It is therefore critical that GPs work in collaboration with interdisciplinary practices so that patients can receive health and social care in an integrated manner. Policy developments and funding schemes should support multidisciplinary team‐based care. Last, evidence regarding GP‐patient relationships is growing and consistently associates strong relationships with better outcomes for patients and the wider health system (i.e., less frequent use of hospital emergency departments, reduced hospital admissions and lower overall healthcare costs).^76^, ^77^ Utilizing a person‐centred approach and developing trusting relationships with patients, including people with disability, should therefore be prioritized in general practice.

There were several gaps in the reviewed literature, including an absence of reporting on healthcare needs and secondary complications of people with disability. While many studies reported utilization of GPs by people with acquired neurological disability and complex needs, only a limited number reported the primary healthcare need presenting to GPs,^42^, ^44^, ^45^, ^48^, ^55^, ^59^ and only two studies reported health problems for which care was required but not received.^42^, ^59^ These findings highlight limited granularity in the available research regarding this population. If effective treatment strategies are to be developed, further research is required to investigate and report commonly presented health needs to GPs, along with health problems that are commonly untreated. It is well established that people with complex disabilities are susceptible to secondary health conditions and comorbidities that can have a substantial negative impact on health and functional capacity^6^, ^7^ and result in referral to emergency departments.^11^, ^22^ This situation is preventable. The current review found a significant barrier to receiving comprehensive healthcare was the limited knowledge of GPs on the secondary health conditions of people with acquired neurological disabilities and complex needs. It is important that future research reports the secondary health conditions and comorbidities of people with acquired neurological disabilities to enhance the knowledge of the general practice workforce.

A rigorous, scoping review methodology using established methods mapped existing literature on health issues faced by adults with acquired neurological disabilities and complex needs, and their utilization of general practice services. The review was comprehensive, including people with a range of acquired neurological disabilities and complex needs (ABI, SCI, MS, CP). However, given findings reflect a range of functional impairments across disabilities, there are limitations with respect to the specific application to a single disability group. The literature included in this review largely reflects studies involving adults and therefore findings may not be applicable to youth or elderly patients. Care should be taken in interpreting findings, as the included studies are drawn from a wide range of settings, each with their own sociocultural setting, health system and funding scheme and a range of demographics (e.g., socioeconomic status, education status and cultural and ethnic backgrounds). Additionally, findings regarding broader healthcare utilization need to be considered within the scope of the included articles which was limited to those reporting general practice service use.

The possibility that all relevant articles may not have been captured through the search strategy is also a risk of any scoping review. Extensive preliminary searches were conducted; however, as the initial review aim was to identify healthcare needs and primary care utilization more generally the final search strategy did not include the terms ‘General Practice’ or ‘Family Physician’. The search captured general practice services under the broad concept headings of primary healthcare (including healthcare needs) and utilization (e.g., of primary healthcare services). Although refinement of review questions is expected in scoping reviews,^31^ it is possible some articles were not captured by the broad search strategy.

## 5 CONCLUSION

This review has highlighted that people with acquired neurological disabilities and complex needs are vulnerable to suboptimal healthcare in general practice. Key factors and implications for practice have been outlined. Findings suggest that high‐quality healthcare for people with acquired neurological disabilities and complex needs requires collaborative relationships and continuity of care across healthcare providers. In addition, evidence suggests the general practice workforce would benefit from further knowledge of disability‐related complications, including mental health concerns. Ongoing policy developments to support multidisciplinary team‐based care are required. Improving the health outcomes of people with acquired disabilities requires that primary health networks have an evidence base to better understand the specific health needs of people with disability and how to meet them. Local solutions are best considered within a co‐design framework that includes GPs and people with disabilities.

## AUTHOR CONTRIBUTIONS

Stacey Oliver, the primary review author, contributed to methodological development, was involved in abstract, title and full‐text screening, the development of the inclusion/exclusion criteria, was responsible for data extraction and data synthesis and formed the original draft and revisions. Jacinta Douglas was responsible for the supervision of the review team, including conceptual and methodological development, oversight of any disagreements relating to publication screening and inclusion and discussion and formulation of concepts and ideas presented within the discussion and also provided review and feedback on the manuscript. Dianne Winkler was responsible for the supervision of the review team, including conceptual and methodological development, oversight of any disagreements relating to publication screening and inclusion and discussion and formulation of concepts and ideas presented within the discussion and also provided review and feedback on the manuscript. Christopher Pearce provided review and feedback on multiple iterations of the manuscript, including interpretation of the findings and ideas presented within the discussion. Ella‐Rose Minter was involved in title, abstract and full‐text screening, assisted with the development of the inclusion/exclusion criteria, contributed to data extraction and provided review and feedback on the manuscript. Hannah K. Jarman implemented the database searches, was involved in title, abstract and full‐text screening, assisted with the development of the inclusion/exclusion criteria, contributed to data extraction and provided review and feedback on the manuscript. Megan Topping was involved in initial method development, designed the review protocol, developed the search strategy and provided review and feedback on the manuscript.

## CONFLICT OF INTEREST

The authors declare no conflict of interest.

## Supporting information

Supporting information.

Supporting information.

## Data Availability

Data sharing is not applicable to this article as no new data were created or analysed in this study.
