# Supplementary material for: The healthcare needs and general practice utilization of people with acquired neurological disability and complex needs: A scoping review
Source: Health Expect. 2022 Nov 2;25(6):2726–45. doi: 10.1111/hex.13640 (PMC9700155; doi:10.1111/hex.13640)
Supplement: Supplementary file 1 — Supporting information. [file HEX-25--s001.docx]

**Appendix A**

| Search strategy |  |
| --- | --- |
| Search concepts | Terms used in database |
| Acquired disability | exp Brain Injuries (MeSH), brain injur*, intracranial injur*, exp Head injuries, penetrating (MeSH), exp Cognition Disorders (MeSH), cogniti* disorder*, cognitive disab*, acquired disab*, communication disab*, severe disab*, profound disab*, exp Multiple Sclerosis (MeSH), multiple sclerosis, exp Cerebral Palsy (MeSH), cerebral palsy, exp Spinal Cord Injuries, spinal cord injur*, ‘stroke (MeSH) AND young adult’ |
| Primary health care | exp Primary Health Care (MeSH), primary health*, primary care,  Community Health Services (MeSH), Community Health (MeSH), Community Care (MeSH), community health*, community care,  “Health Services Needs and Demands” (MeSH), exp Health Service Needs (MeSH), Needs Assessment (MeSH), needs assessment, support needs, service needs, health* needs, care needs, Health Care Costs (MeSH), “Health Care Cost” (Emtree), Health Expenditures (MeSH), health* adj2 cost*, health* expenditure*, exp Pressure Ulcer (MeSH), pressure ulcer, exp Urinary Tract Infections (MeSH), urinary tract infection*, exp Respiratory Tract Infections (MeSH), respiratory tract infection* chest infection*, exp Pneumonia (MeSH), pneumonia, exp Cellulitis (MeSH), cellulitis, exp Seizures (MeSH),seizure, exp Dental Care for Disabled (MeSH), dental care, bladder infection*, exp Eye Infections (MeSH), eye infection*, swallowing difficulties, exp Obesity (MeSH), obesity, exp Overweight (MeSH), overweight, exp Thinness (MeSH), thinness, underweight, weight problems |
| Utilization | exp Health Services (MeSH), Accessibility (MeSH), exp Health Care Utilization (MeSH), access to health*, health* access*, access to care, service* access*, barriers to care, barriers to health*, health care barriers, healthcare barriers, service utili*ation “service use”, healthcare utili*ation, health care utili*ation |
| Databases | MEDLINE, PsycInfo, CINAHL, Scopus, Embase, the Cochrane Library |
