# Supplementary material for: The healthcare needs and general practice utilization of people with acquired neurological disability and complex needs: A scoping review
Source: Health Expect. 2022 Nov 2;25(6):2726–45. doi: 10.1111/hex.13640 (PMC9700155; doi:10.1111/hex.13640)
Supplement: Supplementary file 2 — Supporting information. [file HEX-25--s002.docx]

| **Appendix B** | | |
| --- | --- | --- |
| Exclusion criteria development | | |
|  | Title /Abstract screening | Full text screening |
| Time period | 1. Published before 2010 | 1. Published before 2010 |
| Publication type | 1. In language other than English  2. Articles with no extractable primary data: book chapter, commentary, editorial, conference proceedings, reviews, opinion articles, study protocols | 1. In language other than English  2. Articles with no primary data: book chapter, commentary, editorial, conference proceedings, reviews, opinion articles, study protocols |
| Population | 1. Age 65+ / Mean age 70+  3. Age <18 / Mean age <18 4. Mild disability  5. Developmental, intellectual or learning disability  6. Parkinson’s, Dementia, Alzheimer, Delirium, Huntington’s or epilepsy (without brain injury) 7. Veterans, military or war trauma | 1. Age 65+ / Mean age 70+  3. Age <18 / Mean age <18 4. Mild disability  5. Developmental, intellectual or learning disability  6. Parkinson’s, Dementia, Alzheimer, Delirium, Huntington’s or epilepsy (without brain injury) 7. Veterans, military or war trauma |
| Health care provider | 1. Refer to inpatient facilities or acute/secondary/tertiary care only | 1. Not Family Physician, General Practitioner, Primary Care Physician, Physician in General Practitioners office, Practice Nurse or Nurse Practitioner  2. Secondary/acute or tertiary care e.g., specialist doctor, cardiologist, rheumatologist, urologist, radiologist  3. Does not specify health care provider is Family Physician, General Practitioner, Primary care Physician or Physician in General Practitioners office, Practice Nurse or Nurse Practitioner  4. Does not state or adequately describe setting or health care provider |
| Outcomes | 1. Outcome measure(s) does not include the health care needs or service utilisation of adults with disability and complex needs  2. Reports only on one specific drug or therapy | 1. Outcome measure(s) does not include health care needs or service utilisation of adults with disability and complex needs  2. Reports only on one specific drug or therapy |
| Study Design |  | 1. If includes over 50% of participants over or under 65 years, exclude if groups are not separated by age  2. If includes various disability types, exclude if groups are not separated based on disability type  3. If includes General Practitioner and other health care (e.g., allied health, specialist, inpatient), exclude if groups are not separated by health care providers |
